# Spectral Analysis of a Non-Equilibrium Stochastic Dynamics on a General Network

**DOI:** 10.1038/s41598-018-32650-5

**Published:** 2018-09-25

**Authors:** Inbar Seroussi, Nir Sochen

**Affiliations:** 1 Department of Applied Mathematics, School of Mathematical Sciences, Tel Aviv University, Tel Aviv, 69978 Israel; 2 Sagol School of Neuroscience, Tel Aviv University, Tel Aviv, Israel

## Abstract

Unravelling underlying complex structures from limited resolution measurements is a known problem arising in many scientific disciplines. We study a stochastic dynamical model with a multiplicative noise. It consists of a stochastic differential equation living on a graph, similar to approaches used in population dynamics or directed polymers in random media. We develop a new tool for approximation of correlation functions based on spectral analysis that does not require translation invariance. This enables us to go beyond lattices and analyse general networks. We show, analytically, that this general model has different phases depending on the topology of the network. One of the main parameters which describe the network topology is the spectral dimension $\tilde{{\boldsymbol{d}}}$. We show that the correlation functions depend on the spectral dimension and that only for $\tilde{{\boldsymbol{d}}}$ > 2 a dynamical phase transition occurs. We show by simulation how the system behaves for different network topologies, by defining and calculating the Lyapunov exponents on the graph. We present an application of this model in the context of Magnetic Resonance (MR) measurements of porous structure such as brain tissue. This model can also be interpreted as a KPZ equation on a graph.

## Introduction

Stochastic dynamics on large scale networks has attracted a lot of attention due to its wide occurrence in many disciplines, such as social sciences^1–3^, physics and biology^4–6^, communication and control theory^7^. Also of interest is the population dynamics on networks, which is usually affected by both the topology of the network and some internal stochastic noise in the system. In many applications the network topology is not known and one can only have access to some measurable parameters of the system. Therefore, the analysis of critical phenomena and phase transitions can provide information about the topology of the network^8^. Since the topology of the network is not known, the study of spectral properties of the network is important. For example, the spectra and eigenvectors of the Laplacian of uncorrelated or locally tree-like complex networks reveal interesting properties of the return-probability distribution, the smallest eigenvalue of the Laplacian, as well as localization of the eigenvectors^9,10^. In additions, one can characterize the complexity of the network by analysing the spectral dimension. The analysis of random walks on graphs and their connections to the spectral dimension in equilibrium system is introduced in refs^11–13^.

The main question we ask is what kind of information about the topology of the graph can we extract based on the dynamics of measurable functions, such as the correlation function between the sites of the network in a non-equilibrium model. We show, by working in the spectral domain of the graph, that the spectral dimension plays an important role in determining the dynamical properties of the system.

The model we consider consists of a system of interacting sites on a graph ${\mathscr{G}}$ with *N* vertices and *E* edges between them. We are interested in the stochastic dynamics of some characteristic property ${\{{m}_{i}(t)\}}_{i\in {\mathscr{G}},t\ge 0}$. The property *m*_*i*_(*t*) is linked to a physical measurable quantity in the real world and the graph is the underlying geometry/topology in which the property lives and which usually is a complex network of sites. The model is the following collection of stochastic differential equations in the Stratonovich form on the graph ${\mathscr{G}}$:

1 $$\begin{array}{rcl}\frac{d{m}_{i}(t)}{dt} & = & J\,\sum _{j\in {\mathscr{G}}}\,{W}_{ij}({m}_{j}(t)-{m}_{i}(t))+{g}_{i}(t){m}_{i}(t)\\  & = & -J\,\sum _{j\in {\mathscr{G}}}\,{L}_{ij}{m}_{j}(t)+{g}_{i}(t){m}_{i}(t),\end{array}$$

with the initial condition *m*_*i*_(0) = *m*_0_, where *J* is a constant. The term *g*_*i*_(*t*) is a multiplicative white noise such that

2 $$\langle {g}_{i}(t)\rangle =0,\,\,\,\,\,\langle {g}_{i}(t){g}_{j}(t^{\prime} )\rangle ={\sigma }_{ij}^{2}(t-t^{\prime} ).$$

We choose the Stratonovich form, since its solution is a limiting case of a system with a white noise with a short memory^14^. As a start, we investigate the case where the system does not have any spatial and temporal memory, that is, ${\sigma }_{ij}^{2}(t-t^{\prime} )={\sigma }^{2}{\delta }_{ij}\delta (t-t^{\prime} )$, The topology of the network is encoded in the Laplacian matrix *L*_*ij*_ of the graph ${\mathscr{G}}$, defined as *L*_*ij*_ = *k*_*i*_*δ*_*ij*_ − *W*_*ij*_; then ${k}_{i}={\sum }_{j}\,{W}_{ij}$ is the degree of site *i*. The Laplacian matrix is a zero-row-sum matrix, i.e., ${\sum }_{j}\,{L}_{ij}=0$, for all *i* with non-negative eigenvalues which are ordered by increasing values (assuming *L* is diagonalizable). The graph is assumed to be undirected, with uniformly bounded weights, and with no self loops and multiple edges. Later on, for the asymptotic behaviour analysis, we will add a uniformly-like assumption, which will lead to the relation with the spectral dimension of the graph. The model consists of two parts: an interacting part, where diffusion components exchange with strength depending on their location on the graph, and a non-interacting part, where each component follows a stochastic noise with variance *σ*^2^. The first causes spreading, while the second pushes towards concentration (a.k.a localization or condensation).

**Figure 1:**
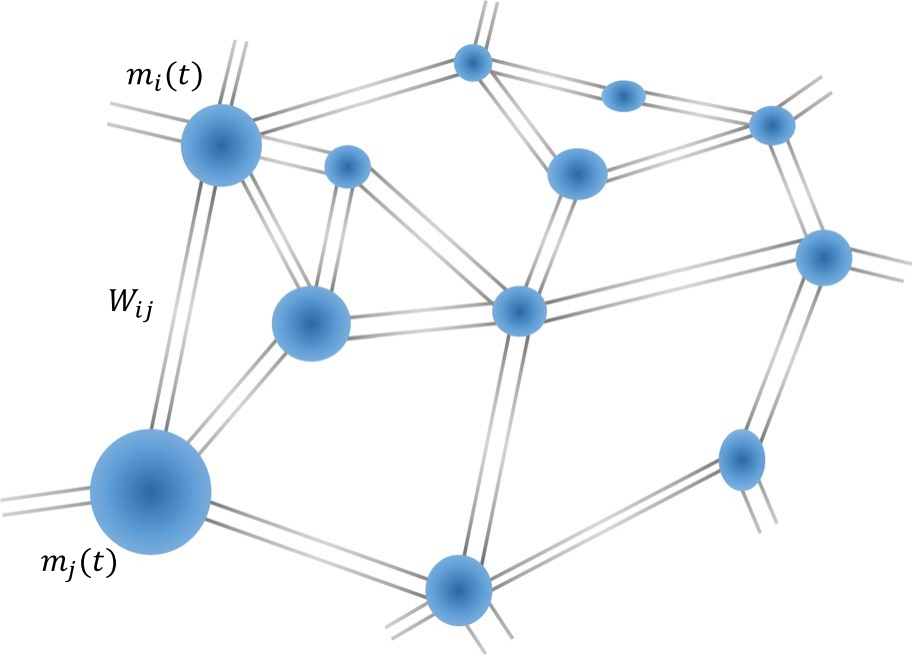
Schematic picture of a network of pores, where *m*_*i*_ is the magnetization in the *i*th pore, and the interaction between the pores is described by the transition matrix *W*.

The model has numerous applications. We present the model in the context of porous systems such as brain tissue which can be measured using Magnetic Resonance Imaging (MRI). The sensitivity of the MR signal to self-diffusion of water molecules can be utilized to extract information about the network describing the topology of cells (neurons) in the brain. The concept of self-diffusion of molecules in a network of pores was already introduced in refs^15–17^. This approach is based on the assumption that diffusion within a pore is much faster than diffusion between pores, which allows one to describe the porous structure as a network of interacting pores. On the measurement time scale the topology is assumed to be fixed. This description goes beyond the traditional models which assume free diffusion of molecules and isolated pores^18–20^. Under this description, *m*_*i*_(*t*) is the magnetization of the *i*th pore at time *t*. The interaction strength *W*_*ij*_ between pores *i* and *j* is influenced by the molecules diffusing between pores. The interaction is time-independent, i.e., possible jamming effects are neglected. In order to model the processes within each pore, we add a stochastic noise coupled to each pore. The variance ${\sigma }_{ij}^{2}$ depends on many parameters, such as temperature, the intrinsic geometry of the pore, fluctuations in the geometry of the pore, and the inhomogeneous magnetic field applied. Figure 1 presents an illustration of a porous medium as a network of pores. This model can be viewed as a generalization of the analysis introduced in ref.^21^. The main challenge in this context is to solve the inverse problem of finding the network topology based on the limited resolution measurements from a single voxel (three-dimensional pixel in an MRI image). A single voxel is a massive average over large domains of around millimetres size, whereas the sizes one is actually interested in are three orders of magnitude smaller of micrometers size, such as radii of axons in the brain. An additional worth noting application of this model is in the context of interface growth; where the property ${m}_{i}(t)={e}^{{h}_{i}(t)}$ can be related to the interface hight, *h*_*i*_(*t*), via the Cole-Hopf transformation. In this representation Eq. (1) is transformed to a discrete KPZ equation on the graph^22^. Exact results have recently been obtained for the one-dimensional case^23,24^.

**Figure 2:**
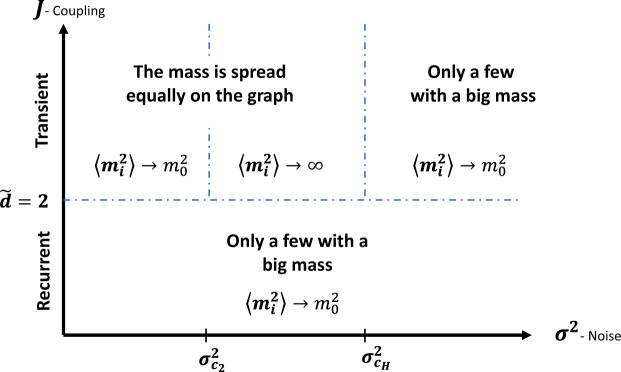
Sketch of the phase diagram of the model.

Our novelty is in analyzing the model on a complex network. The network is not embedded in a lattice topology and therefore does not posses symmetries such as translation invariance. This is important, since in most applications, a lattice topology is highly non-physical. We develop a tool to perform perturbation theory calculations on networks without the usually imposed conservation of momentum constraint. This formalism can be applied to other problems in physics of complex system, quantum disorder, neural science, and more. Using it, we are able to show the existence of a phase transition in our model for spectral dimension $\tilde{d} > 2$. This phase transition is well known on the lattice topology for the Euclidean dimension^25–28^. A sketch of the phase diagram of the model is presented in Fig. 2. It is important to note that the critical values are also geometry/topology dependent as we show later on. Moreover, the connection between this model and brain tissues measured by MRI is also established here for the first time. In the following subsections, we review known results for some simple topologies.

### Mean field topology

We review the results obtained in the mean field topology, i.e., a fully connected graph with constant coupling strength *J* between each two sites. In this topology, one can calculate exactly the equilibrium distribution using the Fokker-Plank equation for a normalized model, i.e., ${M}_{i}(t)={m}_{i}(t)/{\sum }_{i}\,{m}_{i}(t)$, such that ${\sum }_{i}\,{M}_{i}(t)=1$ for any *t*^1^. Under this transformation, all the nodes in the graph are uncorrelated and we can omit the subscript *i*, since the distribution is the same for all the nodes:

$${P}_{{\rm{eq}}}(M)=A\frac{\exp (\,-\,(\mu -\mathrm{1)/}M)}{{M}^{\mu }},$$

where $\mu =1+\frac{2J}{{\sigma }^{2}}$ is a model-dependent parameter, $A=\frac{{(\mu -1)}^{\mu }}{{\rm{\Gamma }}(\mu )}$ is a normalization factor, and Γ(*μ*) is the gamma function. The equilibrium distribution exhibits a Pareto power-law tail. In this case, there are two phase transitions, at *μ* = 1 and at *μ* = 3. This can be understood by deriving the equations for the dynamics of the first and the second moments using the forward Fokker-Plank equation, or alternatively the Feynmann-Kac formula^27^. With this formalism, one can also derive an equation for any *p*th moment.

The steady-state solution for the second moment is, then

3 $${\langle {M}^{2}\rangle }_{\infty }=\frac{\mu -1}{\mu -3}.$$

By comparing these results to Fig. 2, we can identify ${\sigma }_{{c}_{2}}^{2}=1/J$ and ${\sigma }_{{c}_{H}}^{2}\to \infty$. In this case, there are only two phases, and equilibrium is always reached (unless the coupling strength is allowed to be negative). The meaning of these phases in the context of the analysis of porous structure using MRI is as follows: the values *M*_*i*_ describe the density of magnetization in each pore due to the molecules at the *i*th pore at time *t*, and all the pores are connected and interact with pore *i* in the same way. Therefore, one would expect that the contribution of each pore to the magnetization is the same. Here we show that even in the simplified topology of a fully connected graph, the distribution of the magnetization among pores depends on *μ*, which represents the ratio of the interactions strength among pores to the noise variance within each pore. If *μ* < 1 is allowed, then the interpretation is that the contribution to the magnetization comes mainly from a few pores in the long-time limit. This can be regarded as a localization regime which is observed in high magnetic field gradients in the case of isolated pores^15,29^. For 1 < *μ* < 3 the distribution of the molecules among the pores is relatively uniform, but the fluctuations in the magnetization in each pore are very large, whereas above *μ* = 3 the fluctuations become finite for all the pores.

An additional solvable model is the separable model *W*_*ij*_ = *b*_*j*_*k*_*i*_, given that ${\sum }_{j}\,{b}_{j}=1$ and *k*_*i*_ is the degree of the node *i*. Using a similar derivation as in the mean field topology, the critical phases are similar to those in the mean field model (see Supplementary Information Sec. 1).

### Lattice model and other topologies

The lattice topology is studied in various contexts in the mathematics and physics literature. In mathematics, this model is known as the time-dependent Parabolic Anderson Model (PAM), or in its continuum form as the Stochastic Heat Equation (SHE)^30^, and is closely related to the KPZ equation^31^. In the physics literature, one encounters it in many physical systems, such as directed polymers in random media, a case which was analyzed also on trees^2,6^, random interface growth, and turbulence^4^. The discrete lattice case was investigated thoroughly in refs^26,27^, and a generalization with inhomogeneous coupling is given in ref.^25^. In the discrete case, the graph ${\mathscr{G}}$ is embedded in ${{\mathbb{Z}}}^{d}$ space, and with the corresponding Laplacian, the governing stochastic equation reads:

$$\frac{d{m}_{x}(t)}{dt}=\sum _{|x-y|=1}\,{J}_{|x-y|}({m}_{x}(t)-{m}_{y}(t))+{g}_{x}(t){m}_{x}(t);$$

note that in this form the coupling *J*_|*x*−*y*|_ is assumed to be translation invariant. The case where *J*_|*x*−*y*|_ = *J* was analyzed mainly in refs^26,27^ and in the continuum settings, see for example ref.^32^. The analysis in the continuum limit is based on perturbation theory calculations and renormalization group arguments. In this work, we extend this derivation to a general network topology. A generalization to non-constant *J*_|*x*−*y*|_ was done in ref.^25^. An additional variation of the lattice model where the weights *J*_|*x*−*y*|_ are multiplied by a time-dependent random process is introduced in ref.^7^. It is also possible to consider a model in which the noise is not delta correlated (white noise) and it has some correlation form, for example, exponentially decaying with some correlation time, e.g., an Ornstein-Uhlenbeck process. This adds another stochastic equation for the noise in each site. This extension of the model is analyzed in refs^2,25,26,33^.

## Results and Discussion

In this section, we show how the correlation functions provide information about the topology of the underlying graph and the phase transitions of the system. As shown in the methods section (Sec. 4), using perturbation theory we can calculate to all orders the two-point correlation function in the spectral domain. Asymptotic properties of this quantity can be found by analysing the vertex function $\tilde{{\rm{\Gamma }}}(s)$. The vertex function can be expressed in terms of an infinite series of collision matrices ${\tilde{I}}_{ij}(s)={\tilde{P}}_{ij}\ast {\tilde{P}}_{ij}(s)$, where ${\tilde{P}}_{ij}(s)$ is the Laplace transform of the transition probability from site *i* to *j*. Namely,

4 $$\tilde{{\rm{\Gamma }}}(s)=\mathop{\mathrm{lim}}\limits_{L\to \infty }\,\sum _{l=1}^{L}\,\frac{2{\sigma }^{2l}}{{N}^{2}}\,\sum _{{i}_{1}{i}_{l}}\,{[\tilde{I}{(s)}^{l-1}]}_{{i}_{1}{i}_{l}}.$$

The convergence properties of this infinite series depends on the topology of the graph. The information about the graph’s topology is encoded by the collision matrices. In the case of an undirected infinite graph, we assume that the graph is uniformly-like, i.e. the long-time limit of the return probability does not depend on the node location. We can connect the collision matrix in the time domain *I*(*t*) to the spectral dimension, using a property of the Hadamard product (see Supplementary Information Sec. 2):

5 $$\mathop{\mathrm{lim}}\limits_{t\to \infty }\,\frac{\mathrm{ln}\,{\sum }_{k}\,{I}_{ik}(t)}{\mathrm{ln}\,t}=\mathop{\mathrm{lim}}\limits_{t\to \infty }\,\frac{\mathrm{ln}\,{P}_{ii}(t)}{\mathrm{ln}\,t}=-\,\frac{\tilde{d}}{2},$$

where *P*_*ii*_(*t*) is the return probability to the site *i*; its Laplace transform, ${\tilde{P}}_{ii}(s)$, is the generating function of the process. Thus the spectral dimension $\tilde{d}$ is defined by the limit of *P*_*ii*_ (if it exists)^34–37^. It can be understood intuitively as the dimension a random walker “experiences” in a diffusion process on the graph, it measures to what extent the graph is recurrent. In the context of dynamics of molecules, it measures the probability that molecules that started at the *i*th pore will return to this pore. Note that, Eq. (5) refers to the local spectral dimension (for the difference between local and average spectral dimension see ref.^38^). Given Eq. (5), in the limit *s* → 0, we can approximate any row of the matrix $\tilde{I}(s)$ as follows:

6 $$\sum _{{k}_{l}}\,{\mathop{I}\limits^{ \sim }}_{{k}_{l-1}{k}_{l}}(s)=\sum _{{k}_{l}}\,{\mathop{P}\limits^{ \sim }}_{{k}_{l-1}{k}_{l}}\ast {\mathop{P}\limits^{ \sim }}_{{k}_{l-1}{k}_{l}}=\frac{1}{2}\,\mathop{P}\limits^{ \sim }{(\frac{s}{2})}_{{k}_{l-1}{k}_{l-1}}\sim \frac{{B}_{\mathop{d}\limits^{ \sim }}}{2-\mathop{d}\limits^{ \sim }}\,{s}^{-1+\frac{\mathop{d}\limits^{ \sim }}{2}},$$

where ${B}_{\tilde{d}}$ is a constant in *s* (non diverging for $\tilde{d} < 4$). This approximation is used to analyze the behaviour of the vertex function. The analysis of the vertex function is done separately for the two type of topologies: recurrent and transient graphs.

For recurrent graphs, since for each *i*, $\mathop{\mathrm{lim}}\limits_{s\to 0}\,{\tilde{P}}_{ii}(s)=\infty$, the geometric series in Eq. (4) is divergent. Therefore, if a spectral dimension exists, then $\tilde{d}\le 2$ and the vertex function diverges, i.e., all the nodes in the graph are strongly correlated. This corresponds to only one phase of the system in which the magnetization is spread relatively uniformly among the pores.

For transient graphs, since for each *i*, $\mathop{\mathrm{lim}}\limits_{s\to 0}\,{\tilde{P}}_{ii}(s) < \infty$, and under the constraint that

7 $${\sigma }^{2}\parallel \tilde{I}(s){\parallel }_{F}=\frac{{\sigma }^{2}}{2}\,\sum _{i}\,{\tilde{P}}_{ii}(\frac{s}{2})=\sum _{\alpha }\,\frac{{\sigma }^{2}}{s-2J{\lambda }_{\alpha }} < 1,$$

it is possible to take the geometric sum in Eq. (4). Here, *λ*_*α*_ are the eigenvalues of the Laplacian matrix. Note that, in the translation invariant case, an equality in Eq. (7) coincides with the self-consistent equation analyzed in refs^26,27^. The analysis here is at the point where this self-consistent equation breaks down, i.e., close to a phase transition. In the limit of *L* → ∞ the infinite series in Eq. (4) converges to the following function, when plugging in the expression (6) recursively:

8 $$\mathop{{\rm{\Gamma }}}\limits^{ \sim }(s)=\frac{2{\sigma }^{2}}{1-{\sigma }^{2}\frac{{B}_{\mathop{d}\limits^{ \sim }}}{2-\mathop{d}\limits^{ \sim }}\,{s}^{-1+\frac{\mathop{d}\limits^{ \sim }}{2}}}.$$

In the general case, one can write the vertex function in terms of the eigenvalues of the collision matrix:

$$\tilde{{\rm{\Gamma }}}(s)=\tfrac{2{\sigma }^{2}}{N}\,\sum _{i}\,{[{(1-{\sigma }^{2}\tilde{I}(s))}^{-1}]}_{ii}=\tfrac{2{\sigma }^{2}}{N}\,{\rm{Tr}}\,[{(1-{\sigma }^{2}\tilde{I}(s))}^{-1}]=\tfrac{2{\sigma }^{2}}{N}\,\sum _{\alpha }\,\tfrac{1}{1-{\sigma }^{2}{\lambda }_{\alpha }(\tilde{I}(s))},$$

where the ${\lambda }_{\alpha }(\tilde{I}(s))$ are the *α* eigenvalues of the collision matrix $\tilde{I}(s)$.

Renormalization group calculations, similar to the analysis in ref.^32^, show that when $\tilde{d} < 2$ there is only one phase, in which all the nodes in the graphs are strongly correlated, and that when $\tilde{d} > 2$ there exists a critical value ${\sigma }_{{\rm{c}}}^{2}$, which separates between any two phases, one with a finite correlation between two nodes in the network, and the other with infinite correlation. The perturbative calculations, although carried out to all orders, are not capable of capturing the high noise critical point. At the point $\tilde{d}=2$, the RG parameter diverges, which may also indicates that there is a unique point, which we are unable to probe. It may be that a more careful analysis of the asymptotic behaviour of Eq. (2) can lead to a better description of the phases of the system. If we compare the stated results to known results on systems with translation invariance^25^ and to the mean field scenario, the perturbation theory analysis reveals that the system is intermittent for all *σ*^2^ if $\tilde{d}\le 2$, whereas for $\tilde{d} > 2$ the system is intermittent only for ${\sigma }^{2}\le {\sigma }_{{\rm{c}}}^{2}$.

### The graph Lyapunov exponents

The moment Lyapunov exponents are an important tool. A large amount of information about the topology of the system can be extracted by analyzing theses exponents. They are relevant parameters in the context of porous media, since for instance they are closely related to what we can measure using MRI, and can provide insight about the hidden porous structure and complexity. For example, the first Lyapunov exponent can be viewed as a measure of the diffusivity of the medium. In this sub-section, we define and calculate numerically the moment/annealed Lyapunov exponents for simple topologies. The moment Lyapunov exponents can be calculated analytically in the mean field topology^27^. In the lattice topology, one can derive lower and upper bounds^26,27^. The definition of the Lyapunov exponents can be generalized to the case of a general graph topology, where, since the system is not translation invariant and the correlations are node dependent, one needs to average over all the nodes. We define two graph Lyapunov exponents (if the indicated limits exist):

9 $$\hat{{\gamma }_{p}}(L,\sigma )=\mathop{{\rm{l}}{\rm{i}}{\rm{m}}}\limits_{t\to {\rm{\infty }}}\,\frac{\bar{{\rm{l}}{\rm{n}}\,{m}_{p}({\bf{x}},t)}}{t},$$

10 $${\gamma }_{p}(L,\sigma )=\mathop{{\rm{l}}{\rm{i}}{\rm{m}}}\limits_{t\to {\rm{\infty }}}\,\frac{{\rm{l}}{\rm{n}}(\bar{{m}_{p}({\bf{x}},t)})}{t},$$

where ${m}_{p}({\bf{x}},t)=\langle m({x}_{1},t)m({x}_{2},t)\cdots m({x}_{p},t)\rangle$ is the *p*th moment, such that ${\bf{x}}=({x}_{1},{x}_{2},\ldots ,{x}_{p})=(x,x,\ldots ,x)$, where $x\in {\mathscr{G}}$. The overline stands for the average over all the nodes in the graph, e.g., $\bar{{m}_{p}({\bf{x}},t)}=\mathop{{\rm{l}}{\rm{i}}{\rm{m}}}\limits_{N\to {\rm{\infty }}}\,\frac{1}{N}\,{\sum }_{x}\,{m}_{p}({\bf{x}},t)$ and the brackets stands for the average over all possible paths of the random walk up to time *t*. The order of the limits is important. We call the quantity in Eq. (9) the graph sample Lyapunov exponent and the one in Eq. (10) the graph moment Lyapunov exponent. Using Jensen’s inequality, it is clear that $\widehat{{\gamma }_{p}}(L,\sigma )\le {\gamma }_{p}(L,\sigma )$ for any *p* and any graph. In order to examine the behaviour of these exponents, we calculate them numerically for different topologies and noise values. The calculation can be done by solving the equations governing the moments dynamics, which can be derived by using the Stratonovich Fokker-Plank equation for the density of all the nodes *p*(**m**, *t*):

11 $$\frac{\partial \,p}{\partial t}={L}_{{\rm{FP}}}^{\dagger }p=J\,\sum _{i}\,\frac{\partial [({k}_{i}{m}_{i}-{\sum }_{j}\,{W}_{ij}{m}_{j})p]}{\partial {m}_{i}}+\frac{{\sigma }^{2}}{2}\,\sum _{i}\,\frac{\partial }{\partial {m}_{i}}({m}_{i}\frac{\partial ({m}_{i}p)}{\partial {m}_{i}}).$$

Alternatively, one can calculate the moments dynamics based on the backward Fokker-Plank using the Feynmann-Kac formula (see ref.^27^). Since the behaviour of higher moments and the intermittency property are highly dependent on the second moment^26,27^, we focus on the second-moment dynamics for insight into interesting dynamical properties of the model:

12 $$\frac{d\langle {m}_{r}{m}_{l}\rangle }{dt}=2{\delta }_{rl}{\sigma }^{2}\langle {m}_{r}{m}_{l}\rangle -J(\sum _{j}\,{L}_{lj}\langle {m}_{r}{m}_{j}\rangle +\sum _{j}\,{L}_{rj}\langle {m}_{l}{m}_{j}\rangle )={H}_{2}\langle {m}_{r}{m}_{l}\rangle ,$$

where *H*_*p*_ can be identified, as a deterministic Schrödinger operator^26^, in this example, for the second moment *p* = 2. Eqs (11) and (12) are valid for any *σ* as opposed to the perturbative analysis in Secs 2 and 4, which is valid only for small *σ*. Since we do not assume translation invariance in our model, Eq. (12) cannot be reduced to a one-dimensional problem as in the lattice case^26^. Therefore, we use a numerical solution to show the behaviour of the solutions for different graphs. For the numerical calculation, we present Eq. (12) in a vector form, containing all the possible pairs of correlations. The Lyapunov exponents, *γ*_2_ and ${\widehat{\gamma }}_{2}$, are then calculated using the above definitions Eqs (9) and (10) assuming uniform initial conditions. Note that in the lattice case the moment Lyapunov exponents are usually defined in two ways, the first as the long-time limit of a solution to Eq. (12), and the second as the spectral radius of the Schrödinger operator *H*_2_^26,27^. These two definitions are equivalent for the *d*-dimensional lattice and the mean field topologies^26^, but it is not clear whether this is the case for general networks. We believe, following similar arguments as in ref.^26^, that this is the case for all transitive graphs and for graphs of constant degree. We show numerically that it is indeed true for a *d*-regular graph. In general, for a non-trivial topology the spectral radius is a lower bound on the Lyapunov exponent *γ*_2_(*L*,*σ*). An interesting question worth investigating is that of the gap between the two definitions Eqs (9) and (10) with respect to the relation between space (the number of nodes) and time. We compare numerically the two definitions and the largest eigenvalue of the equation *λ*_max_ (see Supplementary Information Sec. 3 for details). We also use as an additional reference the following theoretical lower bound on the second graph moment and sample Lyapunov exponent, where equality is achieved in the mean field:

13 $$\widehat{{\gamma }_{2}}(L,\sigma )\ge 2{\sigma }^{2}-2J\langle k\rangle ;$$

here 〈*k*〉 denotes the average degree of the graph. This bound is proved in refs^26,27^ for the *d*-dimensional lattice case where 〈*k*〉 = 2*d*. The proof for a general graph is similar (see SI Sec. 3). This lower bound shows that for graphs where the average degree distribution (e.g., graphs with power law degree distribution) is diverging, the second graph moment and sample Lyapunov exponent also diverge.

**Figure 3:**
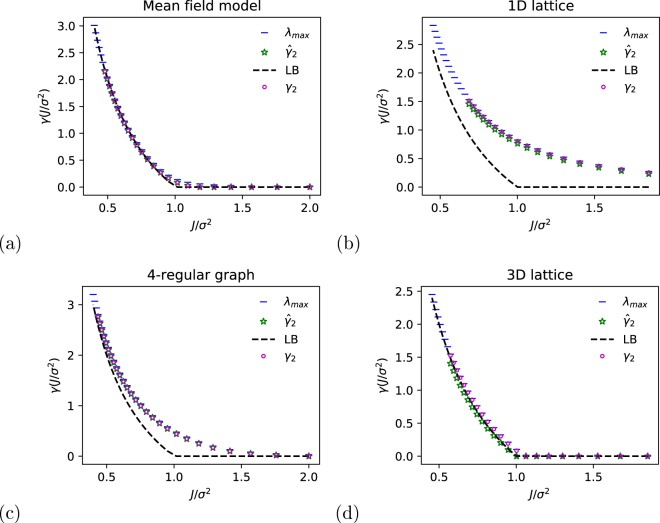
A numerical solution of the graph sample (green star) and moment (purple circle) Lyapunov exponents (Eqs (9) and (10)) and the largest eigenvalue of Eq. (12) (horizontal blue line) for four types of graphs: (**a**) Mean field (**b**) 1D lattice (**c**) regular graph of degree 4 (**d**) 3D lattice. The black dashed line represents the analytical lower bound presented in Eq. (13).

The numerical solution for the mean field Laplacian is presented in Fig. 3a for different values of the ratio *J*/*σ*^2^. Each point was obtained by solving Eq. (12) for different values of the noise variance. The critical point is obtained as expected (Eq. (3)) at *J*/*σ*^2^ = 1. In this case, *γ*_2_ → *λ*_max_ which is also equal to the predicted analytical value Eq. (13) ^27^. Figure 3b,d present the Lyapunov exponents for a 1D and 3D lattice, respectively, with periodic boundary conditions. The simulation shows that, as, predicted by theory^26,27^, in 1D there is no phase transition. On the other hand, in 3D, we see two phase transitions. The two definitions of the Lyapunov exponent Eqs (9) and (10) coincide in these cases. Figure 3c presents the values of the Lyapunov exponent on a *d*-regular graph of degree *d* = 4. In this case, ${\hat{\gamma }}_{2}\to {\gamma }_{2}\to {\lambda }_{{\rm{\max }}}$.

The theoretical analysis for simple topologies, such as the mean field and the lattice model^1,25–27^, suggests the existence of an additional phase transition in the high noise limit in the transient case. This phase transition could not be detected using the perturbative analysis. The numerical solutions in Fig. 3 show some divergence, for high noise values, but it is not clear yet whether this is due to numerical instability or the high noise phase transition.

## Conclusions

We have presented a stochastic model describing diffusion on a graph ${\mathscr{G}}$ with an additional multiplicative stochastic noise. The dependence of the large-scale behaviour of this model on the topology/geometry of the graph is analyzed. The problem we address is whether one can determine the topology of the network based on measurements of some observables. This problem arises in different contexts and disciplines. We show that one of the main structure parameters determining the asymptotic behaviour of the system is the spectral dimension. It is a measure of the complexity of the network which indicates to what extent the network is recurrent. We established the relation between the spectral dimension and the two-point function. This was obtained by calculating the correlation functions perturbatively, in the strength of the noise. This calculation is done in the spectral domain without translation invariance assumption and it shows a direct connection to the return probability distribution. In the transient case, of spectral dimension $\tilde{d} > 2$, there exists a phase transition from a phase when the average correlation between any two nodes is finite and exponentially decaying with time, to a phase in which the average correlation between each two nodes has a power law tail in time. We also present the moment and sample Lyapunov exponents for a general graph. We calculate them numerically in simple topologies. We investigate the connection between the two definitions considered above of the moment Lyapunov exponents on the graph. The connection between these two definitions is an interesting issue and deserves a further analysis. This is also true concerning the connection between the size of the graph and time.

Our analysis and results can be used to study other complex systems, especially, our spectral generalization of the Janssen-De Dominicis technique; using this technique one can calculate moment functions which are related to average observables in networks. In the context of MRI this model is rather a new proposition for a simplified model which takes into accounts the effect of interaction among pores. Our analysis of the correlation functions may lead the way to MRI measurements of the spectral dimension and the Lyapunov exponents. It provides a new way to analyze and understand MR data for porous systems and can lead to new experiments and observable parameters that may reveal exciting structural properties of our brain.

## Methods

### Perturbation theory analysis

The model introduced in Eqs (1) and (2) for a general network is hard to solve exactly. Here, we demonstrate how methods adopted from field theory can provide a unifying framework to produce perturbative approximations for measurable physical quantities. Any stochastic or even deterministic system can be described in terms of a path integral, to which asymptotic methods can be applied systematically. Often of interest are observable quantities such as correlation functions (moments) of **m** or the probability density function of the process *p*(**m**, *t*). Path integral methods provide a convenient tool for computing quantities such as moments and transition probabilities perturbatively using Feynmann diagrams. They also make renormalization group methods (see ref.^32^ Chap. 5) available when perturbation theory breaks down. The strategy of path integral methods is to derive a generating function or functional for the moments and response functions. For stochastic differential equation this can be done using the Janssen-De Dominicis formalism^32,39^. The action derived from Eq. (1) by using this formalism is as follows (see Supplementary Information Sec. 4 for more details). Here, for convenience we write the equation in the equivalent Itô’s form

$$\begin{array}{ccc}{\mathscr{B}}[\mathop{{\bf{m}}}\limits^{ \sim },{\bf{m}}] & = & {{\mathscr{B}}}_{0}[\mathop{{\bf{m}}}\limits^{ \sim },{\bf{m}}]+{{\mathscr{B}}}_{{\rm{i}}{\rm{n}}{\rm{t}}}[\mathop{{\bf{m}}}\limits^{ \sim },{\bf{m}}]\\  & = & \int \,dt\,\sum _{i}\,{\mathop{m}\limits^{ \sim }}_{i}(\frac{{\rm{\partial }}{m}_{i}}{{\rm{\partial }}t}+J\,\sum _{j}\,{L}_{ij}{m}_{j}+\frac{{\sigma }^{2}}{2}{m}_{i})-\frac{1}{2}\,\int \,dt\,\sum _{i}\,{\sigma }^{2}{\mathop{m}\limits^{ \sim }}_{i}^{2}{m}_{i}^{2},\end{array}$$

where the first term is the free theory and the second term is the “interacting” non-quadratic term. Here the initial conditions have been omitted since our focus is on stationary states and a loss of memory of the initial conditions is assumed. The Janssen-De Dominicis formalism allows us to calculate the correlation functions to all orders using perturbation theory by expanding around the non-interaction term. The analysis here is similar to the lattice case^32^, a difference being that we use the eigenbasis of the Laplacian of the graph. The eigenvalues and eigenvectors of the Laplacian operator are defined by the equation *Lϕ*_*α*_ = *λ*_*α*_*ϕ*_*α*_ and ${\sum }_{i}\,{\varphi }_{\alpha }^{\dagger i}{\varphi }_{\alpha ^{\prime} }^{i}={\delta }_{\alpha \alpha ^{\prime} }$, *λ*_*α*_ ≥ 0. The zero eigenvalue corresponds to the uniform eigenfunction: ${\varphi }_{0}^{i}=\frac{1}{\sqrt{N}},\,\forall \,i$. The difficulty in this expansion is that, in contrast to the Fourier eigenfunctions, momentum is not assumed to be preserved, and neither is translation invariance. Therefore, ${\sum }_{\alpha }\,{\varphi }_{\alpha }^{i}{\varphi }_{\alpha }^{j}\ne {\delta }_{ij}$. Keeping this in mind, we can now calculate the correlation function in the Laplacian basis. We define the transforms of the fields with respect to the eigenspace by ${\tilde{m}}_{i}={\sum }_{\alpha }\,{\tilde{m}}_{\alpha }{\varphi }_{\alpha }^{i}$, and by ${m}_{i}={\sum }_{\alpha }\,{m}_{\alpha }{\varphi }_{\alpha }^{\dagger i}$ the inverse transformation: ${\tilde{m}}_{\alpha }={\sum }_{i}\,{\tilde{m}}_{i}{\varphi }_{\alpha }^{\dagger i}$ and ${m}_{\alpha }={\sum }_{i}\,{m}_{i}{\varphi }_{\alpha }^{i}$. The free propagator of the process is diagonal in this basis:

$${\langle {m}_{\alpha }(t){\tilde{m}}_{\alpha ^{\prime} }(t^{\prime} )\rangle }_{0}={\delta }_{\alpha \alpha ^{\prime} }\,\exp \,(\,-\,J{\lambda }_{\alpha }(t-t^{\prime} ))\,{\rm{\Theta }}(t-t^{\prime} )={G}_{0\alpha }(t-t^{\prime} ){\delta }_{\alpha \alpha ^{\prime} }.$$

Let us also introduce the transition probability matrix:

14 $${P}_{ki}(t)=\sum _{\alpha }\,{G}_{0\alpha }(t){\varphi }_{\alpha }^{k}{\varphi }_{\alpha }^{\dagger i}=\sum _{\alpha }\,{\varphi }_{\alpha }^{k}{\varphi }_{\alpha }^{\dagger i}\,\exp \,(\,-\,J{\lambda }_{\alpha }t)\,{\rm{\Theta }}(t)$$

It can be shown, by using similar arguments as for the lattice topology, that $\langle {m}_{\alpha }(t){\tilde{m}}_{\alpha ^{\prime} }(t^{\prime} )\rangle ={\langle {m}_{\alpha }(t){\tilde{m}}_{\alpha ^{\prime} }(t^{\prime} )\rangle }_{0}$, i.e., in this theory the propagator is not renormalized. Note also that due to the symmetric form of the interaction, any correlation function with odd combination of *m* and $\tilde{m}$ vanishes. Therefore an interesting quantity to investigate is the two-point correlation function. We analyze this quantity in the diagonal basis of the Laplacian. The expansion of the two-point correlation function is then written as

$$\begin{array}{c}\langle {m}_{\eta }({t}_{\eta }){m}_{\chi }({t}_{\chi }){\mathop{m}\limits^{ \sim }}_{\rho }({t}_{\rho }){\mathop{m}\limits^{ \sim }}_{\mu }({t}_{\mu })\rangle \\ \begin{array}{ccc} & = & {\langle {m}_{\eta }({t}_{\eta }){m}_{\chi }({t}_{\chi }){\mathop{m}\limits^{ \sim }}_{\rho }({t}_{\rho }){\mathop{m}\limits^{ \sim }}_{\mu }({t}_{\mu })\rangle }_{0}\\  &  & +\,\sum _{l=1}^{{\rm{\infty }}}\,{\langle \frac{1}{l!}{(-{{\mathscr{B}}}_{{\rm{i}}{\rm{n}}{\rm{t}}}[\mathop{{\bf{m}}}\limits^{ \sim },{\bf{m}}])}^{l}{m}_{\eta }({t}_{\eta }){m}_{\chi }({t}_{\chi }){\mathop{m}\limits^{ \sim }}_{\rho }({t}_{\rho }){\mathop{m}\limits^{ \sim }}_{\mu }({t}_{\mu })\rangle }_{0}.\end{array}\end{array}$$

The actions in the basis of the Laplacian reads

$${{\mathscr{B}}}_{0}[\mathop{{\bf{m}}}\limits^{ \sim },{\bf{m}}]=\int \,d\tau \,\sum _{\alpha }\,{\mathop{m}\limits^{ \sim }}_{\alpha }(\tau )({\dot{m}}_{\alpha }(\tau )+J{\lambda }_{\alpha }{m}_{\alpha }(\tau )+\frac{{\sigma }^{2}}{2}{m}_{\alpha }(\tau )),$$

and

$${{\mathscr{B}}}_{{\rm{i}}{\rm{n}}{\rm{t}}}[\mathop{{\bf{m}}}\limits^{ \sim },{\bf{m}}]=-\,\frac{{\sigma }^{2}}{2}\,\sum _{{\alpha }_{1}{\alpha }_{2}{\alpha }_{3}{\alpha }_{4}}\,\int \,d\tau {m}_{{\alpha }_{1}}(\tau ){m}_{{\alpha }_{2}}(\tau ){\mathop{m}\limits^{ \sim }}_{{\alpha }_{3}}(\tau ){\mathop{m}\limits^{ \sim }}_{{\alpha }_{4}}(\tau )\,\sum _{i}\,{\varphi }_{{\alpha }_{1}}^{\dagger i}{\varphi }_{{\alpha }_{2}}^{\dagger i}{\varphi }_{{\alpha }_{3}}^{i}{\varphi }_{{\alpha }_{4}}^{i}.$$

In order to calculate perturbatively the two-point function, we use the concept of the Feynmann diagrams. For convenience, we look at the cumulative function. We sum over all the fully connected diagrams using methods similar to those customarily applied in the lattice case (see Chap. 9 in ref.^32^). The difference here is that conservation of momentum is not assumed. We calculate the two-point cumulative correlation function in the time domain in the eigenbasis of the Laplacian:

$$\begin{array}{c}{\langle {m}_{\eta }({t}_{\eta }){m}_{\chi }({t}_{\chi }){\mathop{m}\limits^{ \sim }}_{\rho }({t}_{\rho }){\mathop{m}\limits^{ \sim }}_{\mu }({t}_{\mu })\rangle }_{{\rm{c}}}\\ \begin{array}{cc} & =\,2\int \,d\tau {G}_{0\rho }(\tau -{t}_{\rho }){G}_{0\eta }({t}_{\eta }-\tau )\,\exp \,(\,-\,\frac{{\sigma }^{2}}{2}{\rm{\Delta }}{t}_{\eta \chi \rho \mu })\,{{\rm{\Gamma }}}_{\eta \chi \rho \mu }(\tau ){G}_{0\mu }(\tau -{t}_{\mu }){G}_{0\chi }({t}_{\chi }-\tau ).\end{array}\end{array}$$

where we define Δ*t*_*ηχρμ*_ = *t*_*η*_ + *t*_*χ*_ − *t*_*ρ*_ − *t*_*μ*_. This function Γ_*ηχρμ*_(*τ*) is called the vertex function in dynamic field theory terms^32^. The vertex function is calculated below using perturbation theory. We show the derivation for the first three orders; the general case follows by induction. The first-order expansion (*l* = 1) yields, using Wick’s theorem,

$${{\rm{\Gamma }}}_{\eta \chi \rho \mu }{(\tau ,\tau )}^{[1]}=2{\sigma }^{2}\cdot \sum _{i}\,{\varphi }_{\mu }^{\dagger i}{\varphi }_{\rho }^{\dagger i}{\varphi }_{\chi }^{i}{\varphi }_{\eta }^{i}.$$

The second-order expansion (*l* = 2) yields

15 $$\begin{array}{rcl}{{\rm{\Gamma }}}_{\eta \chi \rho \mu }{({\tau }_{1},{\tau }_{2})}^{[2]} & = & 2\cdot 2!\cdot {\sigma }^{4}\cdot \exp \,(\,-\,{\sigma }^{2}({\tau }_{2}-{\tau }_{1}))\\  &  & \times \,\sum _{i,j}\,{\varphi }_{\mu }^{\dagger i}{\varphi }_{\rho }^{\dagger i}{\varphi }_{\chi }^{j}{\varphi }_{\eta }^{j}P{({\tau }_{2}-{\tau }_{1})}_{ij}P{({\tau }_{2}-{\tau }_{1})}_{ij}.\end{array}$$

**Figure 4:**
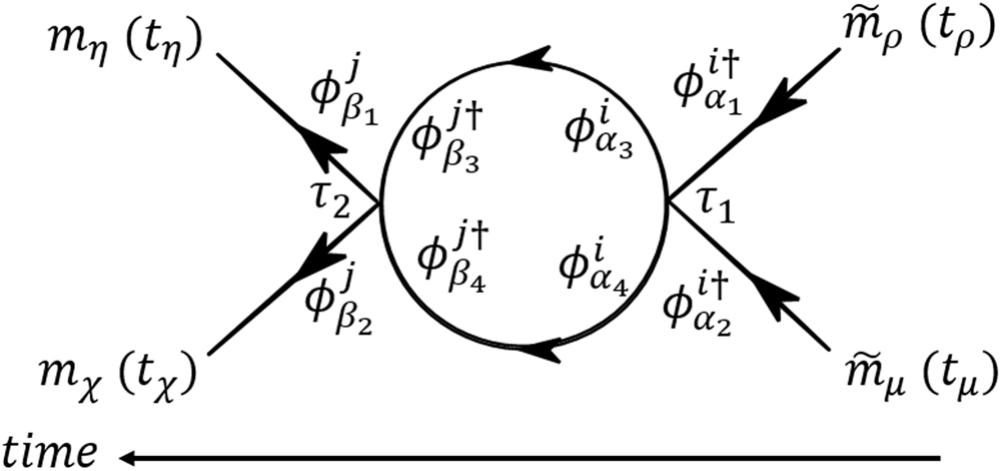
A diagrammatic representation of the second-order in perturbation theory Eq. (15).

Figure 4 shows a diagrammatic representation of the second-order perturbative calculation of Eq. (15) in the eigenbasis domain. The third order expansion (*l* = 3) can derive in the same way:

$$\begin{array}{ccc}{{\rm{\Gamma }}}_{\eta \chi \rho \mu }{({\tau }_{1},{\tau }_{3})}^{[3]} & = & 2\cdot 3!\cdot {\sigma }^{6}\,\exp \,(\,-\,{\sigma }^{2}({\tau }_{3}-{\tau }_{1}))\,\\  &  & \times \int \,d{\tau }_{2}\,\sum _{i,j,k}\,{\varphi }_{\mu }^{\dagger i}{\varphi }_{\rho }^{\dagger i}{\varphi }_{\chi }^{j}{\varphi }_{\eta }^{j}{P}_{ik}({\tau }_{2}-{\tau }_{1}){P}_{kj}({\tau }_{3}-{\tau }_{2}){P}_{kj}({\tau }_{3}-{\tau }_{2}){P}_{ik}({\tau }_{2}-{\tau }_{1}).\end{array}$$

**Figure 5:**
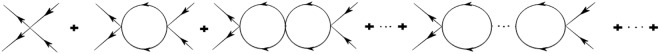
A diagrammatic representation of the sum of geometric series of concatenated two-point loop diagrams (Dyson’s equation); this sum produces the self-consistent equation for the full vertex.

Following this derivation, we can provide the *l*th-order result in perturbation theory:

16 $$\begin{array}{ccc}{{\rm{\Gamma }}}_{\eta \chi \rho \mu }{({\tau }_{1},{\tau }_{L})}^{[l]} & = & 2\cdot l!\cdot {\sigma }^{2l}\,\exp \,(\,-\,{\sigma }^{2}({\tau }_{L}-{\tau }_{1}))\\  &  & \times \,[\sum _{{i}_{1},{i}_{l}}\,{\varphi }_{\mu }^{\dagger {i}_{1}}{\varphi }_{\rho }^{\dagger {i}_{1}}{\varphi }_{\chi }^{{i}_{l}}{\varphi }_{\eta }^{{i}_{l}}\,\prod _{r=2}^{l-1}\,\{\sum _{{i}_{r}}\,{\int }_{{t}_{r}}^{{t}_{r+1}}\,d{t}_{r}I{({t}_{r+1}-{t}_{r})}_{{i}_{r}{i}_{r+1}}\}]\\  & = & 2\cdot l!\cdot \exp \,(\,-\,{\sigma }^{2}({\tau }_{L}-{\tau }_{1}))\\  &  & \times \,\sum _{{i}_{1},{i}_{l}}\,{\varphi }_{\mu }^{\dagger {i}_{1}}{\varphi }_{\rho }^{\dagger {i}_{1}}{\varphi }_{\chi }^{{i}_{l}}{\varphi }_{\eta }^{{i}_{l}}\,{[{\sigma }^{2l}\int d{\tau }_{l}(I\ast I\ast \ldots I)({\tau }_{l}-{\tau }_{1})]}_{{i}_{1}{i}_{l}},\end{array}$$

where we denoted *I*_*ij*_(*t*) = *P*_*ij*_(*t*)*P*_*ij*_(*t*). Note that, in the integration over time we put *t*_2_ = *τ*_1_ and *t*_*l*_ = *τ*_*l*_. Figure 5 shows a diagrammatic representation of the geometric series presented in Eq. (17). Since there is no conservation of momentum, each vertex in the sum has to include a product of four eigenvectors. The function Γ_*ηχρμ*_(*τ*) can be calculated exactly to all orders in perturbation theory, yielding

17 $${{\rm{\Gamma }}}_{\eta \chi \rho \mu }(\tau )=\mathop{\mathrm{lim}}\limits_{L\to \infty }\,\sum _{l=0}^{L}\,\frac{1}{l!}{{\rm{\Gamma }}}_{\eta \chi \rho \mu }{({\tau }_{1},{\tau }_{L})}^{[l]}.$$

The matrix *I*(*t*) is defined as the entry-wise square of the transition matrix *P*(*t*):

$${I}_{ik}(t)={P}_{ik}{(t)}^{2},$$

where *I*(0) = 1. We call the matrix *I*(*t*) the collision matrix. Taking *ρ* = *μ* = *η* = *χ* = 0, and *t*_*ρ*_ = *t*_*η*_ = 0, *t*_*μ*_ = *t*_*χ*_ = *t*, such that ${\varphi }_{\eta }^{\dagger i}={\varphi }_{\chi }^{\dagger j}={\varphi }_{\rho }^{i}={\varphi }_{\mu }^{j}=\frac{1}{\sqrt{N}}$ and applying the Laplace transform $\tilde{{\rm{\Gamma }}}(s)={\int }_{0}^{\infty }\,{\rm{\Gamma }}(t){e}^{-st}dt$, we obtain for the vertex function

18 $$\tilde{{\rm{\Gamma }}}(s)=\mathop{\mathrm{lim}}\limits_{L\to \infty }\,\sum _{l=1}^{L}\,\sum _{{i}_{1}{i}_{l}}\,\frac{2{\sigma }^{2l}}{{N}^{2}}\prod _{r=2}^{l-1}\sum _{{i}_{r}}\,\tilde{I}{(s)}_{{i}_{r}{i}_{r+1}}=\mathop{\mathrm{lim}}\limits_{L\to \infty }\sum _{l=1}^{L}\,\frac{2{\sigma }^{2l}}{{N}^{2}}\,\sum _{{i}_{1}{i}_{l}}\,{[\tilde{I}{(s)}^{l-1}]}_{{i}_{1}{i}_{l}}.$$

In the results section, we present an analysis of the convergence of the vertex function for some graph topologies based on the asymptotic properties of the collision matrix $\tilde{I}(s)$. This allows us to derive the phase diagram of the model.

## Electronic supplementary material

Supplementary Information
