# Supplementary material for: Spectral Analysis of a Non-Equilibrium Stochastic Dynamics on a General Network
Source: Sci Rep. 2018 Sep 25;8:14333. doi: 10.1038/s41598-018-32650-5 (PMC6156338; doi:10.1038/s41598-018-32650-5)
Supplement: Supplementary file 1 — Supplementary Information [file 41598_2018_32650_MOESM1_ESM.pdf]

Supplementary Information: Spectral Analysis of a Non-Equilibrium Stochastic Dynamics on a General Network

Inbar Seroussi[1,*] **and Nir Sochen**[1,2]

[*]corresponding.inbarse@post.tau.ac.il
[1]Department of Applied Mathematics, School of Mathematical Sciences, Tel Aviv University, Tel Aviv 69978, Israel
[2]Sagol School of Neuroscience, Tel Aviv University, Tel Aviv, Israel

ABSTRACT

Unravelling underlying complex structures from limited resolution measurements is a known problem arising in many scientific disciplines. We study a stochastic dynamical model with a multiplicative noise. It consists of a stochastic differential equation living on a graph, similar to approaches used in population dynamics or directed polymers in random media. We develop a new tool for approximation of correlation functions based on spectral analysis that does not require translation invariance. This enables us to go beyond lattices and analyse general networks. We show, analytically, that this general model has different phases depending on the topology of the network. One of the main parameters which describe the network topology is the spectral dimension $\widetilde{d}$. We show that the correlation functions depend on the spectral dimension and that only for $\widetilde{d} > 2$ a dynamical phase transition occurs. We show by simulation how the system behaves for different network topologies, by defining and calculating the Lyapunov exponents on the graph. We present an application of this model in the context of Magnetic Resonance (MR) measurements of porous structure such as brain tissue. This model can also be interpreted as a KPZ equation on a graph.

1 Generalized Mean Field - a separable model

In this section, we analyze a generalized form of the mean field model in which $J_{ij} = k_i b_j$, where $k_i > 0$, and the normalized Laplacian is defined as $L_{ij} = \delta_{ij} - b_j$, with $\sum b_i$=1. This is a generalization of the derivation in Ref. [1]. The model equation, in the Stratonovich form, is as follows:

$$\frac{dM_i}{dt} = J\sum_j b_j M_j - JM_i + \sigma_i g_i(t) M_i$$
$$= J\overline{M(t)}_b - JM_i + \sigma_i g_i(t) M_i.$$

where $g_i(t)$ is a white noise. We assume that all b_j's are positive of order $1/N$. We can define a weighted average magnetization by $\overline{M(t)}_b = \sum_j b_j M_j$. We also introduce $m_i = \frac{M_i}{\overline{M}_b}$, the normalized magnetization with respect to the weighted average magnetization. In order to derive the equation for the normalized magnetization, we derive first the equation for $\overline{M(t)}_b$. For this purpose, we pass to the Itô form:

$$\frac{dM_i}{dt} = J\overline{M}_b - JM_i + \sigma_i g_i(t) M_i + \frac{\sigma_i^2}{2} M_i,$$

If we take the weighted average over all the nodes, and assume that $\sigma_i = \sigma$, then in the limit of $N \to \infty$, using the properties of the white noise, we have

$$\frac{d\overline{M}_b}{dt} = \frac{1}{2}\sigma^2 \overline{M}_b.$$

This allows us to write the Itô equation for the normalized variables using Itô's formula:

$$\frac{dm_i}{dt} = J - Jm_i + \sigma g_i(t) m_i + \frac{\sigma^2}{2} m_i.$$

The corresponding equation in the Stratonovich form reads

$$\frac{dm_i}{dt} = J - Jm_i + \sigma g_i(t) m_i.$$

Next, the the correIsponding Fokker-Planck equation is

$$\frac{\partial p}{\partial t} = -\frac{\partial[(J - Jm_i)\,p]}{\partial m_i} + \frac{1}{2}\sigma^2 \frac{\partial^2}{\partial m_i}\left(m_i \frac{\partial(m_i p)}{\partial m_i}\right). \tag{1}$$

The steady state equilibrium distribution on each node is given by

$$p(m_i) = A m_i^{-(\mu+1)} \exp\left(-\frac{(\mu-1)}{m_i}\right),$$

where we set, $\mu = 1 + \frac{2J}{\sigma^2}$, and A is a normalization constant. Using Eq. (1), we can write the equation for the dynamic of the averages magnetization in the ith pore:

$$\frac{\partial\langle m_i\rangle}{\partial t} = J - J\langle m_i\rangle.$$

The solution to this equation with the initial condition $\langle m_i\rangle(0) = 1$, is $\langle m_i\rangle(t) = (\langle m_i\rangle(0) - 1)\exp(-Jt) + 1$. For the second moment, we have

$$\frac{\partial\langle m_i^2\rangle}{\partial t} = \sigma^2(3-\mu)\langle m_i^2\rangle + 2J\langle m_i\rangle.$$

Therefore, in the steady state the value of the second moment is

$$\langle m_i^2\rangle_\infty = \frac{2J}{\sigma^2(3-\mu)}.$$

The phase transitions then occurs at $\mu = 1$ and $\mu = 3$ as expected.

2 Asymptotic properties of the collision matrix

In this section, we investigate the asymptotic properties of the function $\sum_k I_{ik}(t)$ or of its Laplace transform $\sum_k \widetilde{I}_{ik}(s)$. The matrix $I(t)$ is defined as the entry-wise (Hadamard) product of the transition probability matrix $P(t)$; hence, by a property of the Hadamard product,

$$\sum_k I_{ik}(t) = \sum_k P_{ik}(t)^2 = (P^T P)_{ii}.$$

For an undirected graph, where we assume that the matrix P is symmetric, we have

$$\sum_k P_{ik}(t)^2 = (P^2(t))_{ii} = P_{ii}(2t). \tag{2}$$

Therefore, under the assumption that a spectral dimension exists, we can calculate the limit:

$$\lim_{t\to\infty} \frac{\ln \sum_k I_{ik}(t)}{\ln t} = \lim_{t\to\infty} \frac{\ln P_{ii}(2t)}{\ln t} = \lim_{t\to\infty} \frac{\ln P_{ii}(2t)}{\ln 2t - \ln 2} = \lim_{t\to\infty} \frac{\ln P_{ii}(t)}{\ln t} = -\frac{\widetilde{d}}{2}. \tag{3}$$

Next, we give a stronger statement on the way the function $\sum_k I_{ik}(t)$ behaves in the long-time limit; this is important for the renormalization group calculations. We show under what condition the assumption

$$\sum_k \widetilde{I}_{ik}(s) \sim f(\widetilde{d} - d_c) s^{\frac{\widetilde{d}}{d_c} - 1},$$

is valid. The function $f(\widetilde{d} - d_c)$ may diverge at the point $\widetilde{d} = d_c = 2$. Under the assumption that $P_{ii} \sim t^{-\frac{\widetilde{d}}{2}}$ where the symbol $\sim$ means that

$$C_1 t^{-\frac{\widetilde{d}}{2}} < P_{ii}(t) < C_2 t^{-\frac{\widetilde{d}}{2}} \quad \forall t > t_0, \tag{4}$$

for some constants C_1, C_2, $\widetilde{d}$ and t_0 (in general C_1 and C_2 may allowed to be slowly varying functions as $t \to \infty$). Following the definition of spectral dimension in Ref. [2], where $\widetilde{d}$ is the spectral dimension. This assumption implies, using a Tauberian theorem [2], that

$$C_3 s^{-\gamma} < \left(-\frac{d}{ds}\right)^{\left\lfloor \frac{\widetilde{d}}{2} \right\rfloor} \widetilde{P}_{ii}(s) < C_4 s^{-\gamma}, \tag{5}$$

where $\gamma = \left\lfloor \frac{\widetilde{d}}{2} \right\rfloor - \frac{\widetilde{d}}{2} + 1$, and C_3, C_4 are allowed to be slowly varying functions in s as $s \to 0$. (The spectral dimension is defined through the derivative of $\widetilde{P}_{ii}(s)$ of lowest degree which satisfies the relation Eq. (5)). Knowing this, we can approximate the return probability, for constant C_3 and C_4, e.g., in transitive graphs [3], as

$$\widetilde{P}_{ii}(s) \sim \Gamma(-\frac{\widetilde{d}}{2}+1) s^{\frac{\widetilde{d}}{2}-1} = \frac{B_{\widetilde{d}}}{2-\widetilde{d}} s^{\frac{\widetilde{d}}{2}-1}. \tag{6}$$

Where in the second transition, we separated the divergence in $\widetilde{d} = 2$ by using a property of the Γ-function: $\Gamma(z) = \frac{\Gamma(z+1)}{z}$, such that $\Gamma(-\frac{\widetilde{d}}{2}+1) = \frac{\Gamma(2-\frac{\widetilde{d}}{2})}{-\frac{\widetilde{d}}{2}+1}$ and set $B_{\widetilde{d}} = 2\Gamma(2-\frac{\widetilde{d}}{2})$ a positive number for $\widetilde{d} < 4$, note that $B_{\widetilde{d}}$ diverges in $\widetilde{d} = 4$. To summarize, when there exists a spectral dimension $\widetilde{d}$, Eq. (6) is satisfied, and one can consider the approximation:

$$\sum_k \widetilde{I}_{ik}(s) \sim f(\widetilde{d} - d_c) s^{\frac{\widetilde{d}}{d_c}-1},$$

where $f(\widetilde{d} - d_c) = \frac{B_{\widetilde{d}}}{d_c - \widetilde{d}}$, and $B_{\widetilde{d}}$ is a constant with respect to s and non-diverging for $\widetilde{d} < 2d_c$.

3 Lyapunov exponents on graphs

In this section, we calculate a lower bound on $\gamma_p(J,\sigma)$ and $\widehat{\gamma}_p(J,\sigma)$, defined in the main text Eqs. (9-10). This is a generalization of the derivation in Ref. [4, 5]. We first present the equation for the pth moment on the graph, where $m_p(x_1,\ldots,x_p,t) = \langle m(x_1,t)\cdots m(x_p,t)\rangle$, for $(x_1,\ldots,x_p) \in \{1,N\}$:

$$\begin{aligned}\frac{\partial m_p(x_1,\ldots,x_p)}{\partial t} &= \sum_{l<r}^{p} \delta_{x_l x_r} \sigma^2 m_p(x_1,\ldots,x_p) - J\sum_{r=1}^{p}\sum_{j\in\mathcal{G}} L_{x_r j} m_p(x_1,\ldots,x_p|x_r = j) \\ &= \sum_{l,r=1}^{p} U_{lr}\sigma^2 m_p(x_1,\ldots,x_p|x_r = x_l) - J\sum_{r=1}^{p}\sum_{j\in\mathcal{G}} L_{x_r j} m_p(x_1,\ldots,x_p|x_r = j) = H_p m_p(x_1,\ldots,x_p)\end{aligned} \tag{7}$$

Let us denote $m_p(x_1,\ldots,x_p,t) = V_i(t)$ where ith combination of x_p on the graph $\mathcal{G}$. The size of the vector V is the number of combination of choosing p sites out of N, i.e., $\begin{pmatrix} N \\ p \end{pmatrix}$. The equation can be written in a vector form:

$$\frac{\partial V_i}{\partial t} = \sum_j W_{p,ij}(L,\sigma^2) V_j,$$

where the size of the matrix $W_p(JL,\sigma^2)$ is $\begin{pmatrix} N \\ p \end{pmatrix} \times \begin{pmatrix} N \\ p \end{pmatrix}$. The maximal eigenvalue $\lambda_{\max}$ (the spectral radius) of the matrix $W_p(JL,\sigma^2)$ is a lower bound on the pth Lyapunov exponent $\gamma_p(L,\sigma^2) \geq \lambda_{\max}$ defined in Eq. (13) in the main text. This definition is a generalization of the translation invariant case introduced in Refs. [4, 5]. Note that while, in the lattice topology the annealed Lyapunov exponent (defined in Eq. (5)) is equal to the spectral radius of the Hamiltonian H_p, for a general graph, it is not clear whether this remains true. We show using numerical simulation that for some simple cases, one indeed has $\gamma_p = \lambda_{\max}$.

Now let us give a lower bound on the graph Lyapunov exponent for $p = 2$. We define $n_i(t)$ to be the number of jumps from site i at time t. The probability to stay in site i for infinitesimal time Δt is then,

$$\mathbb{P}(n_i(\Delta t) = 0) = e^{-\Delta t J k_i}.$$

Note that on a general graph this probability is site dependent. The d-dimensional lattice with $k_i = 2dJ$, is an example in which the probability to stay on a site is the same for all the sites. Using the Feynman-Kac formula representing the process in Eqs. (1-2) in the main text, and assuming $m(\mathbf{x},0) = 1$, we can write the second moment as follows:

$$m_2(x_1,x_2,t) = \langle \mathbb{E}_{\mathbf{x}_1,\mathbf{x}_2}\left(e^{\int_0^t\int_0^t g(\mathbf{x}_1,\tau_1)g(\mathbf{x}_2,\tau_2)d\tau_1 d\tau_2}\right)\rangle = \mathbb{E}_{\mathbf{x}_1,\mathbf{x}_2}\left(e^{\frac{1}{2}\langle\int_0^t\int_0^t g(\mathbf{x}_1,\tau_1)g(\mathbf{x}_2,\tau_2)d\tau_1 d\tau_2\rangle}\right)$$
$$= \mathbb{E}_{\mathbf{x}_1,\mathbf{x}_2}\left(e^{\frac{\sigma^2}{2}\int_0^t\int_0^t \delta(\tau_1-\tau_2)\delta(\mathbf{x}_1-\mathbf{x}_2)d\tau_1 d\tau_2}\right) = \mathbb{E}_{\mathbf{x}_1,\mathbf{x}_2}\left(e^{\frac{\sigma^2}{2}\int_0^t \delta(\mathbf{x}_1(\tau)-\mathbf{x}_2(\tau))d\tau}\right),$$

where $x_1, x_2 \in \mathscr{G}$. The first (bracket) average is taken over the stochastic noise and the second is taken over the paths of the random walk. We can bound this from below by looking at the path that stays in the sites x_1 and x_2 up to time t:

$$m_2(x_1,x_2,t) = \langle m(x_1,t)m(x_2,t)\rangle \geq \mathbb{E}_{x_1,x_2}\left(e^{\frac{\sigma^2}{2}\int_0^t \delta(x_1(\tau)-x_2(\tau))d\tau}; n(t)=0\right) = e^{\frac{4\sigma^2}{2}t}\mathbb{E}_{x_1,x_2}\left(n(t)=0\right)$$
$$= e^{2\sigma^2 t}\mathbb{P}\left(n_{x_1}(t)=0\right)\mathbb{P}\left(n_{x_2}(t)=0\right) = e^{2\sigma^2 t - tJk_1 - tJk_2}. \quad (8)$$

Denoting by $\langle k\rangle = \frac{1}{N}\sum_{i=0}^{N} k_i$ the averaging over all the sites, and taking $x_1 = x_2$, one obtains the lower bound on the average Lyapunov exponent:

$$\gamma_2 \geq \widehat{\gamma}_2 = \frac{1}{N}\sum_{x\in\mathscr{G}}\frac{\ln m_2(x,t)}{t} \geq 2\sigma^2 - 2J\langle k\rangle. \quad (9)$$

For a d-regular graph $\langle k\rangle = d$, $\gamma_2(L,\sigma) \geq 2\sigma^2 - 2dJ$. This inequality can be generalized to higher order moments as follows:

$$\gamma_p(L,\sigma) = \frac{1}{N}\sum_{x\in\mathscr{G}}\frac{\ln m_p(x,t)}{t} \geq \frac{p^2\sigma^2}{2} - pJ\langle k\rangle. \quad (10)$$

4 Janssen-De Dominicis response functional

In order to define a "Hamiltonian" for the non-equilibrium system. we use the Janssen-De Dominicis framework. The main idea is to use the identity

$$1 = \int D[\mathbf{m}]\delta\left(\mathbf{m}-\mathbf{m}(\mathbf{g})\right) = \int D[\mathbf{m}]\prod_{i,t}\delta\left(\frac{\partial m_i}{\partial t} + \frac{\delta\mathscr{H}[\mathbf{m}]}{\delta m_i} - g_i(t)m_i - m_0\delta(t-t_0)\right)\det\mathscr{J}[\mathbf{m}]$$
$$= \int D[\mathbf{m}]\int D[\widetilde{\mathbf{w}}]\det\mathscr{J}[\mathbf{m}]\exp\left[-\int dt\sum_i i\widetilde{w}_i\left(\frac{\partial m_i}{\partial t} + \frac{\delta\mathscr{H}[\mathbf{m}]}{\delta m_i} - g_i(t)m_i\right)\right], \quad (11)$$

where we used the Itô form of the equation. We perform the changing of variables $i\widetilde{w}_i \to \widetilde{m}_i$ (note that the integral over $\widetilde{\mathbf{m}}$ is taken on the imaginary axis):

$$1 = \int D[\mathbf{m}]\int D[\widetilde{\mathbf{m}}]\det\mathscr{J}[\mathbf{m}]\exp\left[-\int dt\sum_i \widetilde{m}_i\left(\frac{\partial m_i}{\partial t} - J\sum_j L_{ij}m_j - g_i(t)m_i + \frac{\sigma^2}{2}m_i\right)\right]. \quad (12)$$

The integral is defined using the time discretization $t_l = t_0 + l\tau$,$t_M = t_f$, with the following representation of the measure:

$$D[\mathbf{m}]D[\widetilde{\mathbf{m}}] = \lim_{\substack{\tau\to 0\\ M\to\infty}}\prod_{l=0}^{M}\prod_{\alpha}\frac{-i\tau dm_\alpha(t_l)d\widetilde{m}_\alpha(t_l)}{2\pi}. \quad (13)$$

The presence of the Jacobian $\det\mathscr{J}[m]$ of the transformation is due to the delta function identity $\delta(x-x_0) = \delta(f(x))|\frac{df(x_0)}{dx}|$. In principle one can avoid calculating the Jacobian, since it can be regarded as the normalization of the probability distribution.

This amounts to normalization by tadepole diagrams [6]. The contribution of the determinant in the Itô calculus is 1. Aboe $\widetilde{m}$ is the Martin-Siggia-Rose auxiliary field [7] which is defined on the imaginary axis. In general, we would like to calculate averages of operators $\langle A[m]\rangle_g$:

$$\langle A[\mathbf{m}]\rangle_{\mathbf{g}} \propto \int D[\mathbf{g}]P[\mathbf{g}]A[\mathbf{m}] =$$
$$\int A[\mathbf{m}]\int D[\mathbf{m}]\int D[\widetilde{\mathbf{m}}]\exp\left\{-\int dt\sum_i\left[\widetilde{m}_i\left(\frac{\partial m_i}{\partial t}+\frac{\delta\mathscr{H}[m]}{\delta m_i}\right)\right]\right\}$$
$$\int D[\mathbf{g}]\exp\left\{\int dt\sum_i\left[-\frac{1}{2}\int dt'\sum_j g_i(t)[\sigma_{ij}^2(t-t')]^{-1}g_j(t')+\widetilde{m}_i g_i(t)m_i\right]\right\}.$$

In the second transition, we enter the identity Eq. (11), the integral over g_i, can be calculated using the following identity:

$$\int D[\mathbf{g}]\exp\left[\mathbf{Bg}-\frac{1}{2}g^T[A]^{-1}\mathbf{g}\right]=\sqrt{\frac{(2\pi)^n}{\det A}}\exp\left(\frac{1}{2}(\mathbf{B})^T A^{-1}(\mathbf{B})\right).$$

In our case. we can identify $A_{ij}(t-t')=\sigma_{ij}^2(t-t')$, $B_i(t)=\widetilde{m}_i m_i$, so that

$$\int D[\mathbf{g}]\exp\left\{\int dt\sum_i\left[-\frac{1}{2}\int dt'\sum_j g_i(t)[\sigma_{ij}^2(t-t')]^{-1}g_j(t')+\widetilde{m}_i g_i(t)m_i\right]\right\}$$
$$=\left(\frac{\pi}{\det\sigma^2}\right)^{\frac{n}{2}}\exp\left(-\frac{1}{2}\int dtdt'\sum_{i,j}\widetilde{m}_j(t')m_j(t')\sigma_{ij}^2(t-t')\widetilde{m}_i(t)m_i(t)\right).$$

Since $D[\mathbf{g}]P[\mathbf{g}]=D[\mathbf{m}]P[\mathbf{m}]$, we can define $P[\mathbf{m}]=Z^{-1}\int D[\widetilde{\mathbf{m}}]\exp(-\mathscr{B}[\widetilde{\mathbf{m}},\mathbf{m}])$, where $\mathscr{B}[\widetilde{\mathbf{m}},\mathbf{m}]$ is given by

$$\mathscr{B}[\widetilde{\mathbf{m}},\mathbf{m}]=\mathscr{B}_0[\widetilde{\mathbf{m}},\mathbf{m}]+\mathscr{B}_{\text{int}}[\widetilde{\mathbf{m}},\mathbf{m}],$$

where,

$$\mathscr{B}_0[\widetilde{\mathbf{m}},\mathbf{m}]=\int dt\sum_i\widetilde{m}_i\left(\frac{\partial m_i}{\partial t}+\frac{\delta\mathscr{H}[\mathbf{m}]}{\delta m_i}\right),$$

and

$$\mathscr{B}_{\text{int}}[\widetilde{\mathbf{m}},\mathbf{m}]=-\frac{1}{2}\int dtdt'\sum_{i,j}\widetilde{m}_j(t')m_j(t')\sigma_{ij}^2(t-t')\widetilde{m}_i(t)m_i(t).$$

The partition function is then defined as $Z=\int D[\mathbf{m}]\int D[\widetilde{\mathbf{m}}]\exp(-\mathscr{B}[\widetilde{\mathbf{m}},\mathbf{m}])$. Note that, by averaging over the noise in Eq. (11), it is easy to see that $Z=1$ using the measure given by Eq. (13). Focusing on the delta-correlated noise in space and time, we have

$$\mathscr{B}_0[\widetilde{\mathbf{m}},\mathbf{m}]=\int dt\sum_i\widetilde{m}_i\left(\frac{\partial m_i}{\partial t}+J\sum_j L_{ij}m_j+\frac{\sigma^2}{2}m_i\right), \tag{14}$$

and,

$$\mathscr{B}_{\text{int}}[\widetilde{\mathbf{m}},\mathbf{m}]=-\frac{1}{2}\int dt\sum_i\sigma^2\widetilde{m}_i^2 m_i^2. \tag{15}$$

This formalism allows us to calculate correlation functions between nodes on the graph. For this task, we can use perturbation theory as follows:

$$\langle\prod_{i,k}m_k(t_k)\widetilde{m}_i(t_i)\rangle=\langle\prod_{i,k}m_k(t_k)\widetilde{m}_i(t_i)\sum_{l=1}^{\infty}\frac{1}{l!}(-\mathscr{B}_{\text{int}}[\widetilde{m},m])^l\rangle_0. \tag{16}$$

References

1. Solomon, S. & Richmond, P. Power laws of wealth, market order volumes and market returns. *Phys. A Stat. Mech. Appl.* **299**, 188 – 197 (2001).

2. Hattori, K., Hattori, T. & Watanabe, H. Gaussian field theories on general networks and the spectral dimensions. *Prog. Theor. Phys. Suppl.* **92**, 108–143 (1987).

3. Schonmann, R. H. Multiplicity of phase transitions and mean-field criticality on highly non-amenable graphs. *Commun. Math. Phys.* **219**, 271–322 (2001).

4. Molchanov, S. A. Ideas in the theory of random media. *Acta Appl. Math.* **22**, 139–282 (1991).

5. Carmona, R. & Molchanov, S. A. *Parabolic Anderson problem and intermittency*, vol. 518 (Memoirs of the American Mathematical Society, 1994).

6. Bouchaud, J. P., Cugliandolo, L., Kurchan, J. & Mézard, M. Mode-coupling approximations, glass theory and disordered systems. *Phys. A: Stat. Mech. Appl.* **226**, 243–273 (1996).

7. Martin, P. C., Siggia, E. D. & Rose, H. A. Statistical dynamics of classical systems. *Phys. Rev. A* **8**, 423–437 (1973).
